# Effects of Bovine Lactoferrin on Vaginal Microbiota in Healthy Postmenopausal Women: A Randomized, Double-Blind, Placebo-Controlled Trial

**DOI:** 10.3390/nu18193193

**Published:** 2026-09-27

**Authors:** Chisane Kujirai, Hirotsugu Oda, Miyuki Tanaka, Yayoi Sugimori

**Affiliations:** 1 R&D Division, Food Function Research Institute, Morinaga Milk Industry Co., Ltd., 5-1-83 Higashihara, Zama 252-8583, Kanagawa, Japan; 2 Department of Obstetrics and Gynecology, Bunkyo Garden Women’s Clinic, 2F, 1-1-7 Koishikawa, Tokyo 112-0002, Japan

**Keywords:** lactoferrin, vaginal microbiota, bacterial vaginosis, Nugent score, postmenopausal women

## Abstract

Background/Objectives: This study investigated the effects of bovine lactoferrin (LF) on vaginal microbiota in healthy postmenopausal women. Methods: A randomized, double-blind, placebo-controlled trial was conducted. Healthy Japanese postmenopausal women were administered a placebo or bovine LF (300 mg/day) for 12 weeks. The primary endpoint was the Nugent score and the secondary endpoints were the Nugent sub-scores. The exploratory outcomes included vaginal immunoglobulin and urinary deoxypyridinoline levels. Results: Eighty participants were randomized (placebo, *n* = 40; LF, *n* = 40), of whom 8 were excluded. The remaining 72 participants (placebo group: *n* = 36; LF group: *n* = 36) were included in the per-protocol set. Although the Nugent score was lower in the LF group, the difference was not significant. The *Gardnerella*/*Bacteroides* Nugent sub-score was significantly lower in the LF group than in the placebo group. The vaginal immunoglobulin G1 levels were significantly higher in the LF group than in the placebo group. In addition, the urinary deoxypyridinoline levels were significantly lower in the LF group than in the placebo group. Adverse events were comparable between the groups and no adverse drug reactions were observed. Conclusions: Although oral LF supplementation did not significantly affect the primary endpoint (overall Nugent score), a lower Gardnerella/Bacteroides morphotype score and higher vaginal IgG1 levels were observed. As these findings were derived from secondary and exploratory analyses, respectively, they are considered hypothesis-generating and require confirmation in future studies.

## 1. Introduction

The vaginal microbiota plays a central role in maintaining vaginal homeostasis and supporting women’s health throughout life. In healthy women, the vaginal environment is typically characterized by low microbial diversity and the dominance of vaginally adapted *Lactobacillus* species, which inhibit the growth and colonization of harmful microorganisms. Loss of this *Lactobacillus*-dominant state can lead to bacterial vaginosis (BV) [1,2].

Bacterial vaginosis (BV) is characterized by a polymicrobial community enriched with anaerobic bacteria and is associated with clinical symptoms such as malodor and vaginal discomfort, which negatively affect the quality of life. In addition, BV has been shown to be associated with adverse reproductive outcomes, including infertility [2,3]. As the vaginal environment is strongly influenced by the hormonal status, the decline in estrogen levels associated with menopause reduces glycogen accumulation in the vaginal epithelium, thereby limiting the availability of substrates for *Lactobacillus* species. This leads to reduced lactic acid production, elevated vaginal pH, and the induction of changes that promote a transition toward dysbiotic microbial communities and increase susceptibility to BV [4]. Indeed, BV has been reported in postmenopausal women, with prevalence estimates ranging from approximately 2% to 57% depending on the study population and diagnostic criteria used [4,5].

The Nugent score is widely regarded as the gold standard for the assessment of BV and provides an objective, Gram stain–based evaluation of the vaginal microbiological status. This scoring system is based on the semi-quantitative assessment of three bacterial morphotypes—*Lactobacillus*, *Gardnerella*/*Bacteroides*, and *Mobiluncus*—to generate a composite score. A Nugent score of 0–3 indicates normal flora, 4–6 indicates intermediate microbiota, and 7–10 indicates BV [3,6].

Although the vaginal microbial composition reflects the current balance of microorganisms, mucosal immunity plays an important role in maintaining this balance. Unlike other mucosal sites which have a predominance of immunoglobulin A (IgA), the vaginal mucosa is characterized by a higher proportion of immunoglobulin G (IgG), which is considered to play an important role in local mucosal defense [7]. Immunoglobulins restrict pathogen invasion by neutralizing pathogens and limiting their movement within the mucus layer. Among them, IgG, particularly the IgG1 subclass, has been reported to be involved in protection against pathogens such as human immunodeficiency virus, herpes simplex virus type 2, and *Trichomonas vaginalis* [8,9,10]. Furthermore, recent studies have shown that IgG1 levels decrease in women with BV, suggesting that alterations in mucosal humoral immunity may be associated with vaginal dysbiosis [11]. Taken together, these findings suggest that IgG1 plays an important role in maintaining mucosal immune function in the vaginal environment.

Lactoferrin (LF) is an iron-binding glycoprotein present in mammalian milk and exocrine secretions that is widely used as a health-promoting food ingredient [12]. In addition, LF has been reported to exert antimicrobial and immunomodulatory effects. In vitro studies have shown that bovine LF inhibits the growth of *Gardnerella* species, which play a key role in BV [13]. Clinically, LF has been investigated mainly as an intravaginal formulation in combination with probiotic lactobacilli or in specific populations, such as pregnant women [14]. However, orally ingested LF is unlikely to be absorbed and reach the female genital tract in a form capable of exerting direct antimicrobial activity [15,16]. Furthermore, although several clinical studies have evaluated oral LF supplementation [14], there is a lack of well-designed randomized placebo-controlled trials assessing the effects of LF alone on vaginal microbiota. In particular, to our knowledge, no such evidence is currently available for postmenopausal women, a population at an increased risk of BV.

Menopause-associated estrogen deficiency has also been linked to systemic physiological changes, including increased bone resorption [17], and LF has been reported to influence bone metabolism and has been shown to suppress bone resorption and improve bone-related parameters in ovariectomized models of postmenopausal bone loss [18]. Deoxypyridinoline (DPD) is a widely used urinary biomarker of bone resorption in postmenopausal women [19].

We conducted a randomized, double-blind, placebo-controlled trial to investigate the effects of oral LF intake on vaginal microbiota in healthy postmenopausal women. The primary endpoint was the Nugent score, and the secondary endpoints were the Nugent sub-scores for *Lactobacillus*, *Gardnerella*/*Bacteroides*, and *Mobiluncus* morphotypes. Vaginal immunoglobulin levels and urinary DPD were assessed as exploratory outcomes.

## 2. Materials and Methods

### 2.1. Trial Design and Ethical Approval

This randomized (1:1), double-blind, placebo-controlled trial was conducted in Japan between April and July 2025. The trial was performed in accordance with the current revision of the Declaration of Helsinki [20] and the Ethical Guidelines for Medical and Health Research Involving Human Subjects [21]. The protocol and informed consent forms were reviewed by the Medical Station Clinic Research Ethics Committee on 28 November 2024 (approval number: 241128-3). This trial was registered in the University Hospital Medical Information Network (UMIN) Clinical Trials Registry on 29 November 2024 (registration number: UMIN000056312). This protocol and statistical analysis plan are available in Japanese from the corresponding author upon reasonable request. No participants or members of the public were involved in the design, conduct, report, or dissemination of this study.

### 2.2. Participants

Eligible participants were healthy postmenopausal women aged 50–65 years who had experienced amenorrhea for at least one year. Participants who met any of the following criteria were excluded: diagnosed with BV or bacterial vaginitis within the past three months; a Nugent score ≥ 7 at screening; received antibiotic treatment (oral or intravenous) within the past three months; participants who planned major changes in lifestyle (diet, sleep, exercise, etc.) during the study period; a milk allergy; habitual consumer of LF; receiving treatment for (or with a serious medical history of) hepatic, renal, cardiac, pulmonary, gastrointestinal, hematological, endocrine, or metabolic diseases; a history of drug or severe food allergy; or others who were determined as ineligible for the study by the principal researcher/doctor based on their background, physical observations, and clinical examinations. For the sample size calculation, because no prior studies of oral LF intervention assessing Nugent score improvement were available, estimates were based on similar studies of oral probiotic interventions. The improvement rate in the primary endpoint was assumed to be 16% in the placebo group and 49% in the intervention group [22,23,24]. With a two-sided alpha level of 0.05 and 80% power, the required sample size was estimated to be 37 participants per group for the comparison of the two proportions (Fisher’s exact test) using G*Power version 3.1.9.6 (Heinrich Heine University Düsseldorf, Düsseldorf, Germany). Allowing for potential withdrawals and dropouts, the target sample size was set to 40 participants per group (80 participants in total).

### 2.3. Intervention

After ethics committee approval, the details of the trial were explained to the participants in accordance with the informed consent form, and written informed consent was obtained from all participants. The principal investigator enrolled 80 healthy participants based on background questionnaire and screening results. Participants were randomly allocated to the placebo or LF group. They took three capsules (placebo or 300 mg LF/day) once daily for 12 weeks. Participants were instructed to swallow the capsules with water without chewing. The participants recorded their capsule intake in a diary. Use of treatments, medications, and dietary supplements known to affect the vaginal microbiota or hormonal status, including menopausal hormone therapy, antibiotics, probiotic products, and supplements containing isoflavones or equol, was prohibited during the study period.

### 2.4. Endpoints

The primary endpoint was the Nugent score, which was assessed using vaginal smear samples obtained at baseline (week 0) and after the 12-week intervention (week 12). The secondary endpoints were the Nugent sub-scores (*Lactobacillus*, *Gardnerella*/*Bacteroides*, and *Mobiluncus*). The exploratory outcomes included vaginal IgG1 and IgA, markers of mucosal immune function, and urinary DPD (corrected for urinary creatinine).

### 2.5. Safety Assessment

Any unfavorable and unintended signs, symptoms, or diseases observed during the intervention were defined as adverse events. Adverse events were evaluated according to the Common Terminology Criteria for Adverse Events (CTCAE) version 5.0 [25]. Events in which a causal relationship with the intake of test foods could not be ruled out were designated as adverse drug reactions.

### 2.6. Randomization, Allocation, and Blinding

The test food in the LF group was a hard capsule containing 100 mg of bovine LF. This capsule was filled with bovine LF (MLF-1^®^, Morinaga Milk Industry, Tokyo, Japan), potato starch, silicon dioxide, and calcium stearate. The test food in the placebo group was an identical hard capsule that did not contain LF with the other excipients identical to those in the LF capsule. The appearance, smell, taste, and packaging characteristics of all capsules were similar. An independent allocation manager confirmed that they were indistinguishable at the time of allocation and code breaking. The allocation manager produced a computer-generated allocation table with a block size of four (1:1 ratio) and numbered the test foods consecutively in accordance with the tables. The allocation tables were sealed in opaque envelopes until code breaking. The investigators numbered the enrolled participants and assigned them to each test food item with a corresponding number. The investigators and participants were blinded to code breaking. Code breaking was performed after locking the database and statistical analysis plan.

### 2.7. Vaginal Fluid Sampling and Nugent Score Measurements

Vaginal fluid samples were collected using sterile swabs. The swab was gently inserted into the vaginal canal and rotated several times to collect vaginal secretions. After collection, the swabs were immediately stored under refrigerated conditions and shipped to a testing laboratory (SRL, Tokyo, Japan). The Nugent score and sub-scores of the blinded samples were determined by the testing laboratory according to standard procedures.

### 2.8. Measurement of Vaginal Immunoglobulin G1 and Immunoglobulin A

Vaginal fluid samples were collected using a sterile swab in the same manner as for the Nugent score assessment. The samples were suspended in physiological saline and stored at −80 °C until analysis. Vaginal IgG1 and IgA levels were measured under blinded conditions by an independent testing laboratory (ACEL, Sagamihara, Japan) using a multiplex immunoassay (MILLIPLEX^®^ Human Immunoglobulin Isotyping Panel, Merck, Darmstadt, Germany). The measured values were normalized to the total protein concentration to account for sample variability.

### 2.9. Measurement of Urinary Deoxypyridinoline

The participants were instructed to collect second-morning urine samples (i.e., second urine void after waking) at baseline and week 12. Urinary DPD levels were measured by an independent testing laboratory (LSI Medience Corporation, Tokyo, Japan) using a chemiluminescent enzyme immunoassay and corrected for urinary creatinine levels.

### 2.10. Statistical Analysis

The Nugent score, Nugent sub-scores, vaginal IgG1 and IgA levels (normalized to total protein concentration), and urinary DPD (creatinine-corrected) were analyzed using analysis of covariance, in which the values at week 12 were compared between the groups with adjustment for the corresponding baseline values. Urinary DPD values were log-transformed before analysis because of their skewed distribution. The *Mobiluncus* subscore could not be analyzed using analysis of covariance because of its highly skewed distribution and frequent zero values; therefore, the change from baseline was compared between the groups using the Mann-Whitney U test. Statistical analyses were performed using EZR software version 1.68 (Saitama Medical Center, Jichi Medical University, Saitama, Japan) [26], and statistical significance was set at *p* < 0.05. The primary efficacy analysis was performed in the per-protocol population because the objective of this study was to evaluate the efficacy of bovine lactoferrin under conditions of adequate compliance with the study protocol. Additional analyses in the Full Analysis Set (FAS) were performed to assess the consistency of the findings for all efficacy outcomes except vaginal IgG1 and IgA, which were available only in the per-protocol population. The imputation of missing data was not planned. No adjustment for multiplicity was prespecified for the analyses of secondary and exploratory outcomes in the statistical analysis plan. This manuscript was prepared in accordance with the CONSORT 2025 guideline [27].

## 3. Results

Participants were recruited between 2 December 2024 and 20 February 2025, and 80 provided informed consent. All 80 participants were randomly allocated to two groups (placebo, *n* = 40; LF, *n* = 40). The baseline characteristics of the groups were comparable (Table 1).

After randomization, eight participants were excluded from the per-protocol population (withdrawal of consent, *n* = 1; prohibited concomitant medication use, *n* = 4; noncompliance with study requirements, *n* = 1; ineligibility due to violation of eligibility criteria, *n* = 2). One participant withdrew consent immediately after randomization and provided no post-randomization outcome data. Therefore, the Full Analysis Set comprised 79 participants (placebo, *n* = 39; LF, *n* = 40), whereas the per-protocol population comprised 72 participants (placebo, *n* = 36; LF, *n* = 36) (Figure 1).

Participants began ingesting test foods from 9–21 April 2025 and finished from 1–8 July 2025 (12 weeks). The median (interquartile range) intake rates of the test foods were 100.0% (100.0–100.0%) in the placebo group and 100.0% (99.75–100.0%) in the LF group, with no significant differences.

The Nugent score at week 12 was lower in the LF group than in the placebo group; however, the difference between the groups was not statistically significant (Table 2). Additional analyses in the FAS yielded results that were generally consistent with those obtained in the per-protocol population (Supplementary Table S1).

The *Gardnerella*/*Bacteroides* sub-score was significantly lower in the LF group than in the placebo group at week 12. The *Lactobacillus* and *Mobiluncus* sub-scores were also lower in the LF group than in the placebo group at week 12; however, no significant differences were observed between the groups (Table 3).

Vaginal IgG1 levels at week 12 were significantly higher in the LF group than in the placebo group. Vaginal IgA levels at week 12 were also higher in the LF group than in the placebo group at week 12; however, no significant differences were observed between the groups (Table 4).

In addition, the urinary DPD levels at week 12 were significantly lower in the LF group than in the placebo group (Table 5). Additional analyses in the FAS yielded results that were generally consistent with those obtained in the per-protocol population (Supplementary Table S2).

Adverse events were reported in participants who ingested at least one dose of the test food, and the incidence was comparable between groups (placebo, 20/40 [50.0%]; LF, 16/40 [40.0%]; *p* = 0.50). The reported adverse events included hemorrhoids/anal fissures associated with straining during defecation, knee fractures, visual field narrowing due to glaucoma, and trigger finger due to overuse. No adverse events causally related to the test food were observed.

## 4. Discussion

In this randomized, double-blind, placebo-controlled trial, we examined whether 12-week oral supplementation with bovine LF modulated the vaginal microbiological status in healthy postmenopausal women. The overall Nugent score was lower in the LF group than in the placebo group but did not differ significantly between the groups. Because the prespecified primary endpoint (overall Nugent score) was not significantly different between groups, the findings from secondary and exploratory outcomes should be interpreted as hypothesis-generating. The *Gardnerella*/*Bacteroides* morphotype sub-score was significantly lower in the LF group than in the placebo group. The *Lactobacillus* and *Mobiluncus* morphotype sub-scores did not reach statistical significance. Adverse events were comparable between the groups, and no adverse events with a causal relationship with the test food were identified. The present findings suggest that LF is safe and does not promote the growth of vaginal *Lactobacillus* species, but may suppress the proliferation of *Gardnerella*/*Bacteroides*, which are associated with vaginal dysbiosis in healthy postmenopausal women, although this observation requires confirmation in future studies.

Previous studies on menstruating and pregnant women have suggested that LF acts as a prebiotic that promotes the growth of vaginal *Lactobacillus* species [14]. However, orally administered LF is unlikely to be absorbed and reach the vagina directly [15,16], and many of these studies employed a before-and-after design without an appropriate control group [14,28]. Consequently, the potential influence of probiotic use outside the intervention was not adequately controlled. Furthermore, in many cases, participants received antibiotic treatment before LF administration [14,29,30]. Therefore, it cannot be ruled out that the reported increase in *Lactobacillus* in those studies simply reflected the natural recovery of the indigenous vaginal microbiota, including Lactobacilli, following transient depletion caused by antibiotics. Collectively, the available evidence is insufficient to conclude that LF promotes the growth of vaginal *Lactobacillus* species. To address these concerns, validation in a randomized controlled trial comparing LF alone with placebo is required. Therefore, the present study is, to our knowledge, the first randomized controlled trial to evaluate the effect of LF alone on vaginal microbiota. Postmenopausal women were selected as the study population for two reasons. First, this population allowed us to minimize the influence of hormonal fluctuations associated with the menstrual cycle on vaginal microbiota [2]. Secondly, because the decline in estrogen levels after menopause is associated with a reduction in vaginal *Lactobacillus* populations and therefore an increased risk of BV [4,5], we anticipated that any effects of LF on the vaginal microbiota would be more readily detectable in this population.

Our results showed that LF did not increase *Lactobacillus* abundance but significantly reduced the *Gardnerella*/*Bacteroides* morphotype score. Although increasing vaginal *Lactobacillus* populations is generally considered important for preventing the overgrowth of pathogenic bacteria [2,3,31], such an approach may have limitations in postmenopausal women. In an estrogen-deficient environment, vaginal epithelial glycogen levels are reduced [4], potentially making it difficult for *Lactobacillus* species to establish stable colonization and produce sufficient amounts of lactic acid, even when their abundance is increased.

Hormone replacement therapy is another option for improving the vaginal environment [32]; however, concerns regarding its adverse effects may limit its use in some individuals [33]. In this context, the ability of safe, food-derived ingredients to suppress potentially harmful bacteria without relying on an increase in the *Lactobacillus* population may represent a valuable strategy for maintaining vaginal health. Our results indicate that, in postmenopausal women, oral LF intake alone may suppress undesirable BV-associated bacteria, even when a clear increase in *Lactobacillus* is not observed, although this finding requires confirmation in future studies. The absence of a statistically significant difference in the overall Nugent score may be explained by this response pattern. Since the Nugent score is a composite index that integrates multiple morphotypes [6], a reduction in BV-associated bacteria alone may not result in a marked change in the overall score without a concomitant increase in *Lactobacillus*. In addition, *Mobiluncus* morphotypes were infrequently observed in this trial, limiting its statistical power. However, the lower value in the LF group remained consistent with the suppression of *Gardnerella*/*Bacteroides* morphotypes. Further refinement of the study design may help to elucidate the effects of LF on the overall vaginal microbiota and its potential role in promoting vaginal health.

Because orally ingested LF is unlikely to be absorbed and reach the vaginal mucosa and exert direct antibacterial activity [15,16], suppression of *Gardnerella*/*Bacteroides* morphotypes is unlikely to be attributable to the intravaginal antibacterial activity of LF. However, LF has been reported to influence immune function and inflammatory responses, and clinical studies have suggested that oral LF intake modulates systemic inflammatory and immune-related markers [34,35]. These effects may create conditions that are unfavorable for BV-associated bacterial proliferation. To explore the mechanism by which LF suppresses potentially harmful vaginal bacteria, we measured immunoglobulins in vaginal fluid using exploratory analyses. As a result, we observed a significant increase in the IgG1 levels. Therefore, the increase in the vaginal IgG1 levels observed in the present study may reflect an improvement in the local immune environment of the genital mucosa. One possible explanation is that IgG1 produced by B cells stimulated by LF in the intestinal immune system was transported to the vaginal mucosa via the circulation [36,37]. Alternatively, LF-stimulated B cells might have migrated to the female reproductive tract and produced IgG1 there [38]. However, the present study does not establish a causal relationship between increased vaginal IgG1 levels and the reduction in Gardnerella/Bacteroides morphotype scores. Nevertheless, the observed increase in vaginal IgG1 levels together with the reduction in Gardnerella/Bacteroides morphotype scores raises the possibility that vaginal mucosal immunity may be involved in the observed microbiological findings. This hypothesis may help explain the reduction in Gardnerella/Bacteroides morphotype scores without a concomitant increase in Lactobacillus abundance; however, further mechanistic studies are required to evaluate this possibility. Overall, our results suggest that, in postmenopausal women, LF may not act as a prebiotic that increases the vaginal *Lactobacillus* population.

Although evaluated exploratorily, we observed a reduction in urinary deoxypyridinoline (DPD) by LF. Because DPD is influenced not only by estrogen deficiency but also by immune and inflammatory processes, the reduction in DPD might have been due to the suppression of osteoclast-mediated bone resorption by LF [17]. The beneficial effects of LF on bone health have been demonstrated in numerous ovariectomized animal models, and these effects have been suggested to be associated, at least in part, with the anti-inflammatory properties of LF [18,39,40]. However, the evidence in humans remains limited. Previous clinical studies have evaluated LF in combination with ribonucleases, making it difficult to determine whether LF alone contributes to the observed benefits [41,42]. Therefore, it remains unclear whether LF has a favorable effect on human bone health, and the present findings are the first to suggest that LF alone may contribute to the maintenance of bone health in postmenopausal women.

Limitations of the present study were related to the postmenopausal life stages of the study population. A decline in vaginal *Lactobacillus* dominance has been commonly reported after menopause, and the baseline microbial milieu differs substantially from that in premenopausal women [4,43]. Accordingly, the effects of LF may vary according to the life stage, and extrapolation of the present findings to other populations should be performed with caution. Furthermore, participants were healthy postmenopausal women without BV at enrollment and exhibited low baseline Nugent scores. Therefore, the present findings do not establish the efficacy of LF for the prevention or treatment of BV, nor do they demonstrate improvements in clinically meaningful vaginal symptoms. Further studies in menstruating nonpregnant and pregnant women are required to determine whether the proposed mechanism is broadly applicable to different physiological conditions. In addition, the primary efficacy analyses were prespecified to be conducted in the per-protocol population. Although additional analyses in the Full Analysis Set yielded generally similar results, potential bias arising from post-randomization exclusions cannot be completely excluded. Additionally, further studies are required to confirm the exploratory findings on vaginal immunoglobulins and urinary DPD. Because no adjustment for multiplicity was prespecified for the analyses of secondary and exploratory outcomes, the observed findings should be interpreted cautiously because type I error inflation cannot be excluded.

## 5. Conclusions

The present study showed that 12 weeks of oral LF supplementation did not significantly reduce the overall Nugent score in postmenopausal women. However, a lower Gardnerella/Bacteroides morphotype score and higher vaginal IgG1 levels were observed. As these findings were derived from secondary and exploratory analyses, respectively, they are considered hypothesis-generating and require confirmation in future studies.

## Figures and Tables

**Figure 1:**
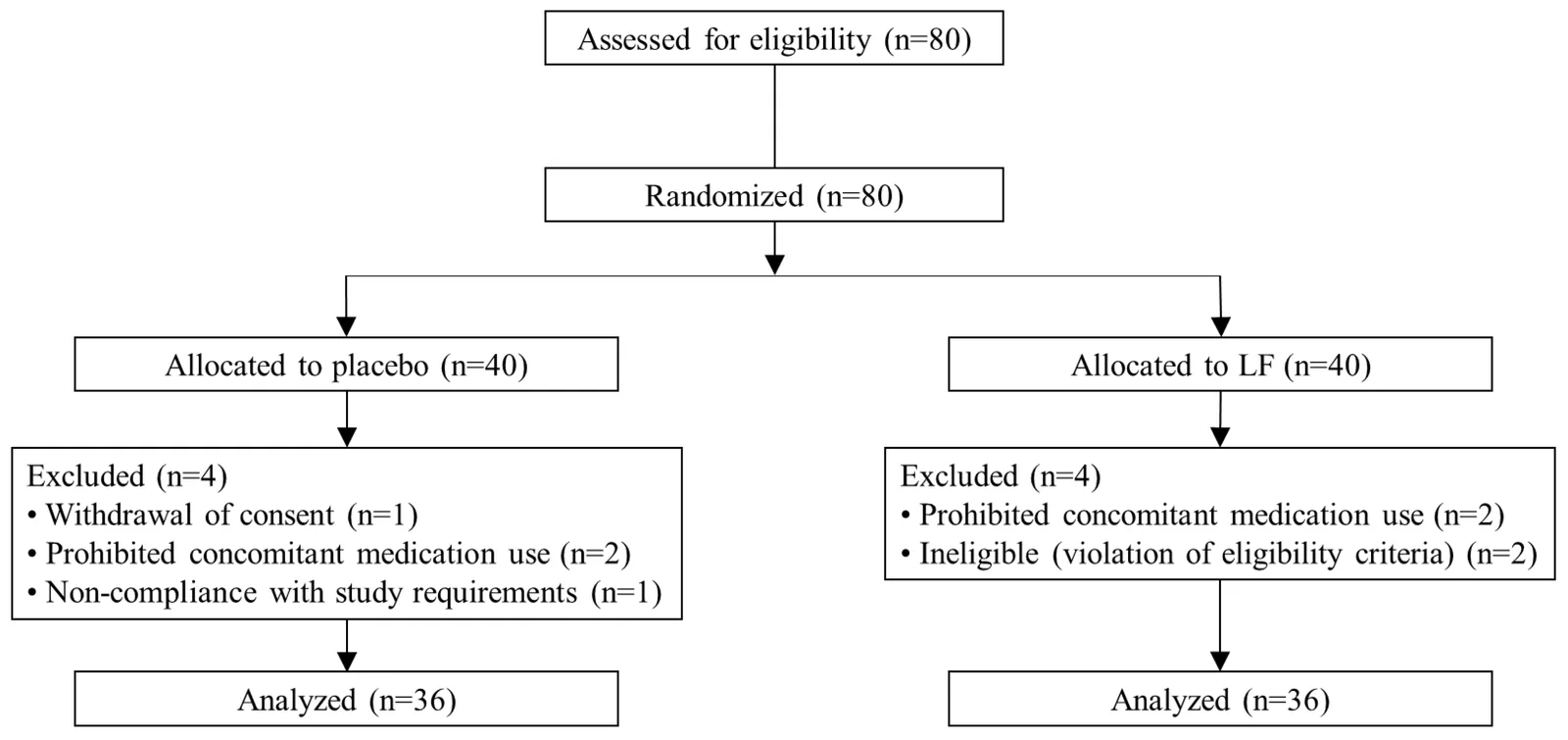
CONSORT flow diagram of the study.

**Table 1:** Baseline demographics.

	Placebo	LF
Participants, *n*	40	40
Age in years	56.1 (3.8)	55.0 (3.5)
(range)	(50–63)	(50–64)
Height (cm)	158.4 (5.0)	157.5 (5.5)
Weight (kg)	55.6 (8.2)	55.2 (9.1)
BMI (kg/m^2^)	22.1 (3.2)	22.2 (3.3)
Nugent score	2.0 (2.0)	2.0 (2.0)
*Lactobacillus*	1.3 (1.1)	1.4 (1.2)
*Gardnerella*/*Bacteroides*	0.7 (1.5)	0.7 (1.4)
*Mobiluncus*	0.0 (0.0)	0.0 (0.0)

Values are expressed as means (standard deviation). BMI, body mass index; LF, lactoferrin.

**Table 2:** Nugent score.

		Placebo (*n* = 36)	LF (*n* = 36)	Difference (95% CI)	*p* Value
Nugent score	Week 0	2.111 (0.349)	1.972 (0.330)		
	Week 12	2.389 (0.383)	1.972 (0.302)	−0.386 (−1.342 to 0.571)	0.424
	Δ12 weeks	0.278 (0.494)	0.000 (0.350)		

Values are reported as means (standard error). Difference represents the adjusted between-group difference (LF minus placebo) with 95% confidence interval (CI). The *p*-value is the placebo vs. LF. LF, lactoferrin.

**Table 3:** Nugent sub-scores.

		Placebo (*n* = 36)	LF (*n* = 36)	Difference (95% CI)	*p* Value
*Gardnerella*/*Bacteroides*	Week 0	0.778 (0.252)	0.639 (0.226)		
	Week 12	0.972 (0.277)	0.250 (0.146)	−0.708 (−1.334 to −0.081)	0.027
	Δ12 weeks	0.194 (0.365)	−0.389 (0.237)		
*Mobiluncus*	Week 0	0.000 (0.000)	0.000 (0.000)		
	Week 12	0.028 (0.028)	0.000 (0.000)		-
	Δ12 weeks	0.028 (0.028)	0.000 (0.000)	0.000 (0.000 to 0.000)	0.331
*Lactobacillus*	Week 0	1.333 (0.187)	1.333 (0.195)		
	Week 12	1.389 (0.253)	1.722 (0.297)	0.333 (−0.378 to 1.045)	0.353
	Δ12 weeks	0.056 (0.249)	0.389 (0.274)		

Values are reported as means (standard error). Difference represents the adjusted between-group difference (LF minus placebo) with 95% confidence interval (CI). The *p*-value is the placebo vs. LF. LF, lactoferrin.

**Table 4:** Vaginal Immunoglobulin G1 and Immunoglobulin A levels.

		Placebo (*n* = 36)	LF (*n* = 36)	Difference (95% CI)	*p* Value
IgG1 (ng/μg protein)	Week 0	11.682 (1.060)	17.190 (1.612)		
	Week 12	13.740 (1.292)	19.330 (1.675)	4.500 (0.064 to 8.936)	0.047
	Δ12 weeks	2.058 (1.541)	2.140 (2.086)		
IgA (ng/μg protein)	Week 0	9.447 (1.600)	10.157 (1.377)		
	Week 12	14.574 (2.395)	15.873 (3.606)	0.741 (−7.360 to 8.843)	0.856
	Δ12 weeks	5.126 (2.515)	5.716 (3.160)		

Values are reported as means (standard error). Difference represents the adjusted between-group difference (LF minus placebo) with 95% confidence interval (CI). The *p*-value is the placebo vs. LF. IgA, immunoglobulin A; IgG1, Immunoglobulin G1; LF, lactoferrin.

**Table 5:** Urinary deoxypyridinoline levels.

		Placebo (*n* = 36)	LF (*n* = 36)	Difference (95% CI)	*p* Value
DPD [Ln (nM/mM Cr)]	Week 0	1.972 (0.052)	2.007 (0.033)		
	Week 12	2.126 (0.075)	1.933 (0.043)	−0.207 (−0.374 to −0.040)	0.016
	Δ12 weeks	0.154 (0.078)	−0.074 (0.046)		

Values are reported as means (standard error). Difference represents the adjusted between-group difference (LF minus placebo) with 95% confidence interval (CI). The *p*-value is the placebo vs. LF. Cr, creatinine-corrected; DPD, deoxypyridinoline; LF, lactoferrin; Ln, natural log-transformed.

## Data Availability

The data supporting the findings of this study are available from the corresponding author upon reasonable request.

## Supplementary Materials

The following supporting information can be downloaded at: https://www.mdpi.com/article/10.3390/nu18193193/s1, Table S1: Nugent score and sub-scores in the Full Analysis Set.; Table S2: Urinary deoxypyridinoline levels in the Full Analysis Set.
