# Supplementary material for: Effects of Bovine Lactoferrin on Vaginal Microbiota in Healthy Postmenopausal Women: A Randomized, Double-Blind, Placebo-Controlled Trial
Source: Nutrients. 2026 Sep 27;18(19):3193. doi: 10.3390/nu18193193 (PMC13666833; doi:10.3390/nu18193193)
Supplement: Supplementary file 1 [file nutrients-18-03193-s001.zip › nutrients-4541880-supplementary.pdf]

**Table S1.** Nugent score and sub-scores in the Full Analysis Set.

| | | Placebo (n=39) | LF (n=40) | Difference (95% CI) | P value |
|---|---|---|---|---|---|
| Nugent score | Week 0 | 2.026 (0.328) | 2.025 (0.319) | | |
| | Week 12 | 2.359 (0.376) | 2.225 (0.319) | −0.134 (−1.092 to 0.825) | 0.782 |
| | ∆ 12 weeks | 0.333 (0.484) | 0.200 (0.338) | | |
| *Gardnerella/Bacteroides* | Week 0 | 0.718 (0.235) | 0.675 (0.222) | | |
| | Week 12 | 1.000 (0.270) | 0.400 (0.175) | −0.593 (−1.334 to −0.081) | 0.066 |
| | ∆ 12 weeks | 0.282 (0.350) | −0.275 (0.229) | | |
| *Mobiluncus* | Week 0 | 0.000 (0.000) | 0.000 (0.000) | | |
| | Week 12 | 0.077 (0.057) | 0.000 (0.000) | | - |
| | ∆ 12 weeks | 0.077 (0.057) | 0.000 (0.000) | 0.000 (0.000 to 0.000) | 0.155 |
| *Lactobacillus* | Week 0 | 1.308 (0.177) | 1.350 (0.184) | | |
| | Week 12 | 1.282 (0.241) | 1.825 (0.277) | 0.519 (−0.158 to 1.195) | 0.131 |
| | ∆ 12 weeks | −0.026 (0.237) | 0.475 (0.263) | | |

Values are reported as means (standard error). Difference represents the adjusted between-group difference (LF minus placebo) with 95% confidence interval (CI). The P-value is the placebo vs LF. LF, lactoferrin

**Table S2.** Urinary deoxypyridinoline levels in the Full Analysis Set.

| | | Placebo (n=39) | LF (n=40) | Difference (95% CI) | P value |
|---|---|---|---|---|---|
| DPD [Ln (nM/mM Cr)] | Week 0 | 1.971 (0.048) | 2.006 (0.038) | | |
| | Week 12 | 2.088 (0.073) | 1.940 (0.041) | −0.163 (−0.322 to −0.004) | **0.045** |
| | Δ 12 weeks | 0.117 (0.076) | −0.066 (0.043) | | |

Values are reported as means (standard error). Difference represents the adjusted between-group difference (LF minus placebo) with 95% confidence interval (CI). The P-value is the placebo vs LF.
Cr, creatinine-corrected; DPD, deoxypyridinoline; LF, lactoferrin; Ln, natural log-transformed
